# Levodopa responsiveness and white matter alterations in Parkinson's disease: A DTI‐based study and brain network analysis: A cross‐sectional study

**DOI:** 10.1002/brb3.2825

**Published:** 2022-11-24

**Authors:** Juncong Du, Xuan Zhou, Yi Liang, Lili Zhao, Chengcheng Dai, Yuke Zhong, Hang Liu, Guohui Liu, Lijuan Mo, Changhong Tan, Xi Liu, Lifen Chen

**Affiliations:** ^1^ Department of Neurology The Second Affiliated Hospital of Chongqing Medical University Chongqing People's Republic of China

**Keywords:** brain network, levodopa responsiveness, Parkinson's disease, white matter

## Abstract

**Background:**

Patients with Parkinson's disease (PD) present various responsiveness to levodopa, but the cause of such differences in levodopa responsiveness is unclear. Previous studies related the damage of brain white matter (WM) to levodopa responsiveness in PD patients, but no study investigated the relationship between the structural brain network change in PD patients and their levodopa responsiveness.

**Methods:**

PD patients were recruited and evaluated using the Unified Parkinson's Disease Rating Scale (UPDRS). Each patient received a diffusion tensor imaging (DTI) scan and an acute levodopa challenge test. The improvement rate of UPDRS‐III was calculated. PD patients were grouped into irresponsive group (improvement rate < 30%) and responsive group (improvement rate ≥ 30%). Tract‐based spatial statistics (TBSS), deterministic tracing (DT), region of interest (ROI) analysis, and automatic fiber identification (AFQ) analyses were performed. The structural brain network was also constructed and the topological parameters were calculated.

**Results:**

Fifty‐four PD patients were included. TBSS identified significant differences in fractional anisotropy (FA) values in the corpus callosum and other regions of the brain. DT and ROI analysis of the corpus callosum found a significant difference in FA between the two groups. Graph theory analysis showed statistical differences in global efficiency, local efficiency, and characteristic path length.

**Conclusion:**

PD patients with poor responsiveness to levodopa had WM damage in multiple brain areas, especially the corpus callosum, which might cause disruption of information integration of the structural brain network.

## 1 INTRODUCTION

Parkinson's disease (PD) is the second most common neurodegenerative disorder that affects 2**–**3% of the population aged ≥65 years (Goldman & Postuma, 2014). Though treating PD with levodopa is strongly recommended, approximately 20% of PD patients are poorly responsive to levodopa (Rizek et al., 2016). Moreover, the subtypes of PD, including the postural instability and gait difficulty (PIGD) subtype, the tremor‐dominant subtype, and the mixed subtype, are related to patients’ responses to levodopa (Jankovic et al., 1990). Among the three subtypes, PD patients of PIGD subtype are less responsive to levodopa compared to those of tremor‐dominant subtype (Jankovic et al., 1990). The specific causes and mechanisms of this difference in levodopa responsiveness between PD patients are still unclear. Therefore, we performed this study on PD patients of PIGD subtype.

Previous diffusion tensor imaging (DTI) studies have reported that PD patients presented white matter (WM) damage, the majority of them reported widespread microstructural WM damage in terms of reduced fractional anisotropy (FA), increased mean diffusivity (MD), axial diffusivity (AD), and radial diffusivity (RD) in many tracts, including corticospinal tract, optic radiation, corpus callosum, frontal and occipital tracts, nigrostriatal tract, inferior frontooccipital fasciculus, and uncinate fasciculus and posterior thalamic radiation (Pozorski et al., 2018; Rau et al., 2019; Zhang et al., 2016). These changes reflect different pathological changes in WM of PD patients. It is reported that PIGD patients exhibited severe motor symptoms and WM degradation than patients of tremor‐dominant subtype, mainly in the association tracts linking the anterior and posterior parts of the brain (Wen et al., 2018). Moreover, some parts of WM (e.g., splenium/body/genu of the corpus callosum, bilateral cingulum) have been associated with levodopa responsiveness (Guan et al., 2019; Li et al., 2018; Zhou et al., 2021). These studies either compared levodopa responsiveness‐related WM alterations between PD patients with different subtypes or adopted only a single analytical method for DTI research. More importantly, these studies reached no consistent conclusion, while the related brain WM network impairment has not been investigated yet. In this study, we further explored the relationship between WM changes and levodopa responsiveness with four different analytical methods, and analyzed the brain WM network for a more comprehensive understanding.

DTI is a non‐invasive neuroimaging technique that is able to detect changes in WM microstructural properties in living patients (Le Bihan, 2003). Tract‐based spatial statistics (TBSS), automating fiber‐tract quantification (AFQ), deterministic tracing (DT) and region of interest (ROI) analyses are commonly used methods to investigate the integrity of WM based on DTI. There are various parameters, including FA, MD, AD, and RD. In general, aging and disease processes are expected to produce reductions in FA due to axonal degeneration and demyelination, increases in MD and RD due to loss of tissue boundaries, and an increase in AD as a possible result of higher directional coherence among remaining axons (Alexander et al., 2007; Madhyastha et al., 2014). Therefore, DTI is a useful method to investigate WM change in PD patients with different levodopa responsiveness.

WM damage may disrupt the brain network and affect the function of the brain (Hilal et al., 2021). Recently, analysis based on graph theory has been used extensively to study the organization of human brain networks and their change in diseases (Reijneveld et al., 2007). Previous studies have identified WM damage in PD patients using DTI, which disrupts the brain network of PD patients (Abbasi et al., 2018; Kamagata et al., 2017). Meanwhile, a few studies have documented levodopa responsiveness‐related WM damage in PD patients (Zhou et al., 2021), which we speculate that WM damage may affect the brain network of PD patients underlying the mechanism of levodopa unresponsiveness. Additionally, some functional connectome studies have also revealed that degeneration of nigrostriatal neurons in PD may be associated with a reorganization of a functional large‐scale network (Berman et al., 2016; Gao et al., 2017). Such reorganization may also exist in the structural network of PD patients. However, structural brain network damage in PD patients and its relationship with levodopa responsiveness has seldom been researched.

In this study, we measured WM alterations using TBSS, AFQ, DT, and ROI analyses based on DTI in PIGD PD patients with different levodopa responsiveness. We also analyzed the topological characters in the structural brain network between PIGD PD patients with different levodopa responsiveness, aiming to provide insights into the roles of WM damage and structural brain network disruption in PD and their effect on levodopa responsiveness, which may assist the research, understanding, and treatment of PD.

## 2 MATERIALS AND METHOD

### 2.1 Ethics statement

This study protocol was approved by the Ethics Committee of The Second Affiliated Hospital of Chongqing Medical University (2017‐56), and written consent was obtained from each patient. This study was performed in accordance with the Declaration of Helsinki.

### 2.2 Subjects

We recruited PIGD PD patients from the Department of Neurology, The Second Affiliated Hospital of Chongqing Medical University, China, from January 2017 to December 2020, who were diagnosed according to the Movement Disorder Society (MDS) clinical diagnostic criteria for PD (Postuma et al., 2015). Data, including age, sex, duration of PD, use of anti‐Parkinson drugs, and detailed medical history, were collected. Disease severity was assessed by two experienced neurologists using the Unified Parkinson's Disease Rating Scale (UPDRS) (Goetz et al., 2008) and the Hoehn and Yahr Scale (H&Y) (Hoehn & Yahr, 1967). Patients of the PIGD subtype were defined based on the ratio of mean tremor score (items II‐16, III‐20, and III‐21) and mean PIGD score (items II‐13, II‐14, II‐15, III‐29, and III‐30) less than or equal to 1.0 (Merello et al., 2002). The Mini‐Mental State Examination (MMSE) was used to screen for cognitive dysfunction. The exclusion criteria were as follows: (1) suffering from neurological diseases other than PD; (2) presenting with psychiatric disorders; (3) significant cognitive dysfunction (MMSE < 20); (4) unable to accept DTI scan and UPDRS scoring, and (5) obvious WM change on T1‐ or T2‐weighted imaging.

### 2.3 Acute levodopa challenge test

Patients were pretreated with 60 mg of domperidone at least 72 hours before the test to prevent side effects. The UPDRS‐III score was evaluated before the acute levodopa challenge test. For drug‐naive patients, the levodopa dose is 250 mg levodopa/benserazide in the morning and in the fasting state (Albanese et al., 2001). For patients under chronic treatment, all anti‐Parkinson drugs were withdrawn for 12 hours and an overnight fast was observed before the evaluation day (Albanese et al., 2001). Long‐acting dopamine agonists were stopped 72 hours prior to evaluation day (Albanese et al., 2001). Suspension containing levodopa/benserazide 1.5 times the individual morning levodopa dose and 100 ml water was administrated for hastening drug absorption in the morning of the evaluation day (Albanese et al., 2001). The UPDRS‐III score was evaluated again one hour after levodopa administration. The improvement rate was calculated as follows: (UPDRS‐III score before levodopa – UPDRS‐III score after levodopa challenge)/(UPDRS‐III score before levodopa challenge). The patients were divided into two groups: responsive group (improvement rate ≥ 30%) and irresponsive group (improvement rate < 30%) (Merello et al., 2002).

### 2.4 MRI scanning

MRI scanning was performed 48 hours to 24 hours before the acute levodopa challenge test using a 3.0T MRI scanner (PHILIPS‐2E80B50, Philips, Amsterdam, Holland). All subjects were at a comparable baseline with no treatment. Considering the possible effect of tremors on MRI scanning, we prescribed sedatives for temporary control of tremors in patients when necessary. High‐resolution T1‐weighted 3D anatomical images were obtained in the sagittal orientation using a fast field echo sequence, repetition time (TR) = 7.4 ms; echo time (TE) = 3.6 ms; flip angle = 8°; field of view (FOV) = 250×250 mm^2^; matrix = 228×227; 150 slices; slice thickness = 1.1 mm with no gap and voxel size = 1.1×1.1×1.1 mm^3^. DTI parameters were a spin echo‐based echo‐planar imaging sequence in contiguous axial planes that included 16 volumes with diffusion gradients applied along 15 noncollinear directions (*b* = 800 s/mm^2^) and one volume without diffusion weighting (*b* = 0 s/mm^2^). Each volume consisted of 65 contiguous axial slices covering the entire brain (TR = 6973 ms; TE = 75 ms; flip angle = 90°; FOV = 224 × 224 mm^2^; matrix = 112×112 and voxel size = 2 × 2 × 2 mm^3^). Notably, due to the limitations of scanning equipment, an acquisition protocol of 15 noncollinear directions with *b* = 800 s/mm^2^ was chosen. This is a relatively suitable, but not ideal, acquisition protocol based on the limitations of the equipment. Therefore, the accuracy of tractography in our study should be considered judiciously.

### 2.5 DTI processing

Digital imaging and communications in medicine files of DTI were converted into NIFTI format and then processed using FMRIB Software Library (FSL) software (FSL 5.0.9). Eddy currents and head motion were corrected using affine registration to the first b0 image using the "eddy_correct" and "fdt_rotate" functions in FSL. A brain extraction tool was then used to generate a binary brain mask from the b0 image. To provide a multiple‐aspect view of WM alterations, the diffusion tensors were linearly fitted to the diffusion‐weighted images using the "dtifit" tool in FSL to generate maps of FA, MD, AD, and RD.

### 2.6 TBSS

The images were then skull‐stripped and FA, MD, AD, and RD images were calculated using the DTIFIT function for all participants. Image analysis using TBSS included the following steps: (1) nonlinear alignment of all subjects’ FA images into a common space using the FMRIB nonlinear registration tool; (2) affine‐transformation of the aligned images into standard MNI152 1 mm space; (3) averaging of the aligned fractional anisotropy images to create a 4D mean FA image; (4) thinning of the mean FA image to create a mean FA "skeleton" that represents the centers of all WM tracts common to the group, and (5) thresholding of the FA skeleton at fractional anisotropy 0.2 to suppress areas of extremely low mean FA and exclude those with considerable interindividual variability. (6) Voxelwise statistical analysis of the WM skeleton was performed using Randomise‐FSL's nonparametric permutation inference tool (5000 permutations), with threshold‐free cluster enhancement (TFCE) for multiple comparison correction, and age, sex, baseline UPDRS‐III score, challenged UPDRS‐III score, MMSE score, and H&Y stage were adjusted as covariates. Additionally, we also analyzed whether there were regions in which the metrics presented a positive or negative relationship with improvement rate using linear regression according to the manual of FSL (https://fsl.fmrib.ox.ac.uk/fsl/fslwiki/GLM). In voxelwise statistical analysis, TFCE‐corrected *p* < .05 was considered statistically significant. The Johns Hopkins University White Matter Tractography Atlas was added to FMRIB Software Library to describe the identified regions.

### 2.7 AFQ

For fiber tract identification, we used automated fiber quantification (AFQ) software (Yeatman et al., 2012) to identify 20 WM tracts in each participant's brain. The identification procedure included three primary steps: (1) whole‐brain deterministic fiber tractography, (2) waypoint ROI‐based tract segmentation, and (3) probability map‐based fiber refinement. Twenty fiber tracts were identified according to the predefined ROIs and probability maps: bilateral thalamic radiation, corticospinal tract (CST), cingulum cingulate, cingulum hippocampus, callosum forceps major, callosum forceps minor, bilateral inferior front‐occipital fasciculus (IFOF), bilateral inferior longitudinal fasciculus (ILF), superior longitudinal fasciculus (SLF), bilateral uncinate, bilateral arcuate. Then, each fiber bundle was divided into 100 segments. Finally, the FA, MD, AD, and RD of each segment of each fiber bundle were calculated. The comparison method of AFQ data between the two groups was formulated after improvement according to previous literature reports (Sun et al., 2015).

### 2.8 Corpus callosum analysis

Because AFQ could not track and analyze the whole corpus callosum, we further analyzed the corpus callosum using two different ways: (1) DT: Diffusion Toolkit software (version 0.6.4.1) was used to estimate the diffusion tensors. Fiber tracking was based on the fiber assignment by continuous tracking (FACT) algorithm with a FA threshold of 0.15 and angle threshold of 45 degrees; a single ROI defined around the corpus callosum on a midsagittal slice was drawn manually to track (Catani & Schotten, 2008). Then, the estimates of the FA, MD, RD, and AD average values were automatically calculated by the software and registered for each subject. (2) Atlas‐based ROI analysis: the corpus callosum was mapped using the JHU DTI‐based white‐matter atlases, then, the ROI were registered into the native brain space of each subject to calculate FA, MD, AD, and RD metrics.

### 2.9 Brain network construction

For Network Construction, we used a MATLAB‐based open‐source software PANDA (Cui et al., 2013). Nodes and edges are the two fundamental elements of a network. In this study, we constructed a structural network using the following procedures: (1) removing the basal ganglia regions of the automated anatomical labeling 116 templates (Rolls et al., 2020) to make template A; (2) the atlasing of the basal ganglia (Keuken et al., 2017; Keuken et al., 2013), including striatum, external globus pallidus, globu pallidus interna, red nucleus, substantia nigra, and subthalamic nucleus were assembled, and the overlapped parts were removed to make template B; (3) adding template A and template B, numbering each brain region. Finally, we got a brain template containing 124 brain regions (see atlas of 124 brains and ROI list of 124 brains in the Supporting Information). Individual T1‐weighted images were co‐registered to b0 images in DTI native space using an affine transformation. The converted T1 images were normalized to a T1 template in MNI space through nonlinear transformation. The 124‐region template was used to distort the MNI space into the DTI native space through inverse transformation. Then diffusion MRI tractography was performed, and all tracts in the DTI dataset were computed by seeding each voxel with an FA > 0.2. The tractography was terminated if it turned an angle >45 degrees or reached a voxel with an FA < 0.2. As a result, all fiber pathways between the 124 ROIs in the brain were constructed using deterministic tractography method. For each pair of ROIs *i* and *j*, we defined the number of connected fiber streamlines (FN) with two endpoints located in these two regions as the weight of the edge between ROIs *i* and *j*. Therefore, for each participant, an FN‐weighted 124×124 structural connectivity matrix was constructed. FA‐weighted structural connectivity matrix was constructed in the same way (mean matrixes are shown in Figure S1).

### 2.10 Brain network analysis

Graph theory and network‐based statistics (NBS) were applied to analyze the structural brain network. All topological parameters were calculated using the GRETNA toolbox (Wang et al., 2015). The specific parameters were as follows: the sign of the matrix was positive, the thresholding method was the value of the matrix element; for the FN‐weighted matrix, the threshold sequence was 3, and for the FA‐weighted matrix the threshold sequence was 0.2. Both the FN‐weighted matrix and FA‐weighted matrix were weighted network types. The random network number was 100.

To investigate the integration of the structural brain network, we explored global efficiency (*E*_g_), and characteristic path length (*L*_p_). Segregation was assessed by the local efficiency (*E*_loc_) and clustering coefficient (*C*_p_). The local topological properties (betweenness centrality, degree centrality, nodal clustering coefficient, nodal efficiency, and nodal local efficiency) were obtained to assess the centrality of a node. The small‐worldness was used to determine the efficiency of global integration and segregation of information flow between nodes of the network. Briefly, normalized path length (λ) can be used as a surrogate of global integration, and normalized clustering coefficient (γ) can be used as a surrogate of global segregation. Small‐worldness (σ) in a network exists if λ ≈ 1 and γ ≫ 1, and suggests optimal information sharing between the local (γ) and distant neighbors (λ) of a node.

The NBS approach was utilized to detect abnormalities of edge connective strength using the NBS software package (Zalesky et al., 2010). This process consists of four steps. First, independently test the hypothesis of interest at every connection in the network. Second, choose a primary test statistic threshold. Third, identify subnetworks among the set of suprathreshold connections using a breadth or depth search. Finally, compute the NBS‐corrected *p*‐value for each component using permutation testing.

### 2.11 Statistical analysis

#### 2.11.1 Demographics and clinical variables

SPSS version 19.0 (IBM, Armonk, USA) was used for statistical analysis. Quantitative data with normal distribution were expressed as mean ± standard deviation (SD) or were expressed as median (25th percentile, 75th percentile). The rank‐sum test was used for the H&Y stage comparison between groups. Sex distribution between both groups was analyzed using the χ^2^ test. Independent *t*‐test was used for age, baseline UPDRS‐III score, UPDRS‐III score after acute levodopa challenge, and improvement rate comparison.

#### 2.11.2 White matter alterations

Voxelwise statistical analysis of the WM skeleton was performed using Randomise, FSL's nonparametric permutation inference tool (5000 permutations), with TFCE correction for multiple comparisons. In voxelwise statistical analysis, TFCE‐corrected *p* < .05 was considered statistically significant. Independent *t*‐test was used for the FA, MD, AD, and RD of corpus callosum fiber constructed by deterministic track, and Cohen's *d* was calculated. The FA, MD, AD, and RD of the main 20 fiber tracts constructed by AFQ between the responsive group and irresponsive group were compared using permutation tests with false discovery rate (FDR) correction. *p* < .05 was considered statistically significant.

#### 2.11.3 Brain network statistical analysis

Statistical analyses of global and nodal parameters were performed with the GRETNA toolbox. Between the two groups, the global network parameters and nodal network parameters of each node were compared by a two‐sample *t*‐test with FDR correction for multiple comparisons. *p* < .05 was considered statistically significant. Both positive and negative correlations between edge connective strength (defined by the FN and FA) in the two groups were explored using the nonparametric permutation statistic (5000 permutations; *p* < .001) with NBS correction.

## 3 RESULTS

### 3.1 Demographics and clinical characteristics

**FIGURE 1:**
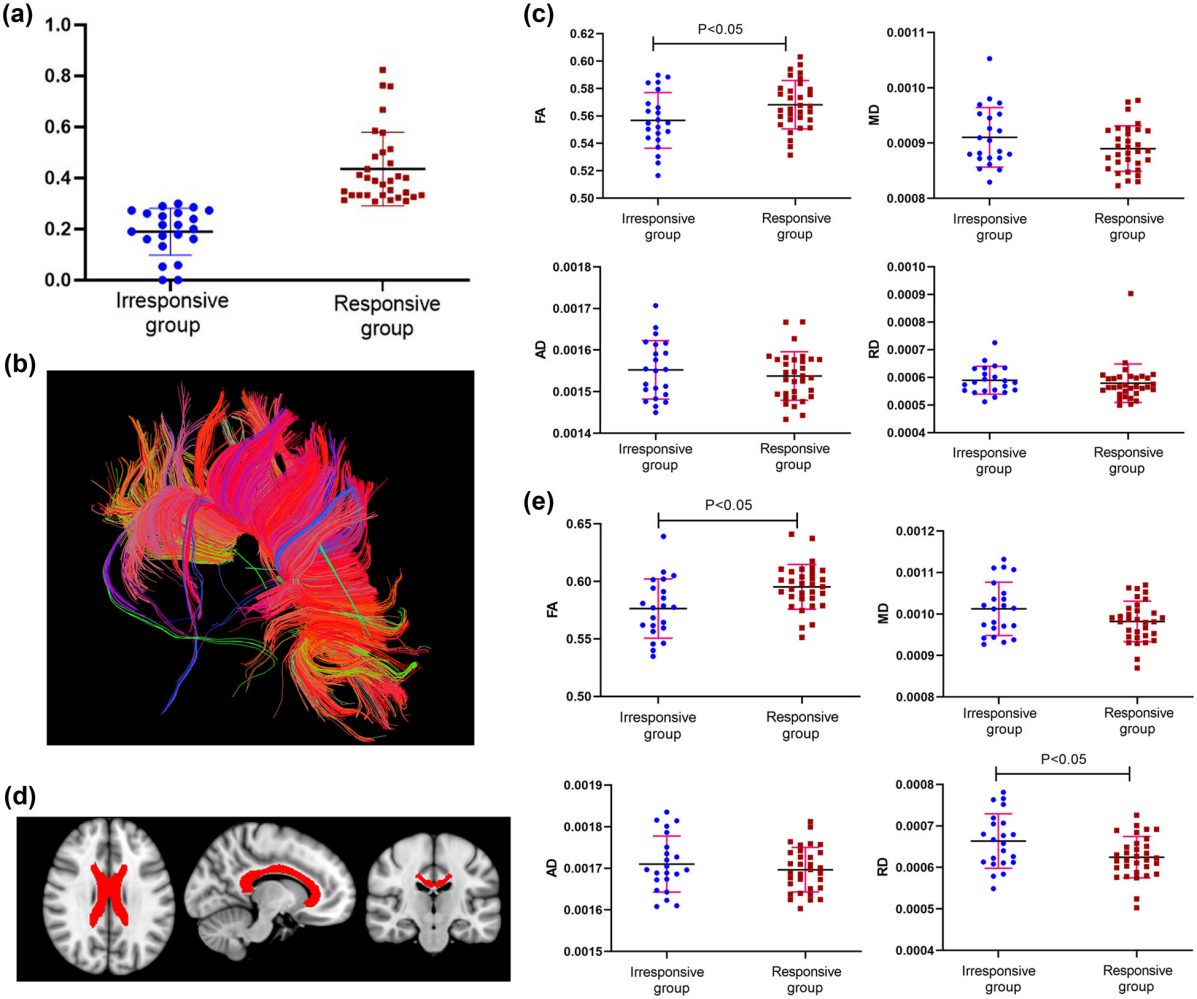
(A) Box plots of improvement rate after acute levodopa challenge test between the two groups. (B) Representative image of corpus callosum fibers of one subject by deterministic tracing (DT). (C) Group differences in FA/MD/AD/RD of corpus callosum based on DT. (D) Region of interest (ROI) of corpus callosum used in ROI analysis. (E) Group differences in FA/MD/AD/RD of corpus callosum based on ROI analysis. *p < .05

A total of 54 PD patients (25 females; mean age, 66.87 ± 8.644 years; MMSE, 25.15 ± 3.5; H&Y, 2 (1.5, 2.5)) participated in this study. There were 22 patients in the irresponsive group and 32 patients in the responsive group. The ratio of mean tremor score and mean PIGD score of all patients was less than 1.0, and the mean ratios of the two groups are (0.31 (0.10, 0.63) vs. 0.31 (0.10, 0.63), *p* = .911). No significant difference was detected between the irresponsive group and responsive group in age (68.91 ± 7.43 vs. 65.47 ± 9.235 years, *p* = .153) and sex (female 45% vs. 46.8%, *p* = .57). There was no significant difference between the irresponsive group and responsive group in MMSE score (25.09 ± 3.21 vs. 25.18 ± 3.74, *p* = .922), H&Y stage (2.0 (1.75, 2.5) vs. 2.0 (1.5, 2.5), *p* = .45), baseline UPDRS‐III score (26.41 ± 11.35 vs. 33.72 ± 16.12, *p* = .073) and UPDRS‐III score after acute levodopa challenge test (21.50 ± 10.73 vs. 17.91 ± 7.32, *p* = .149). Improvement rate (0.19 ± 0.09 vs.0.44 ± 0.14, *p* < .001) significantly differed between the irresponsive and the responsive groups (Table 1 and Figure 1).

**TABLE 1:** Demographics and clinical characteristics of Parkinson's disease patients

	All	Irresponsive group	Responsive group	*p*‐values
Number	54	22	32	NA
Age (years)	66.87 ± 8.644	68.91 ± 7.43	65.47 ± 9.235	.153
Sex (female/male)	25/29	10/12	15/17	.57
UPDRS‐III (baseline)	30.74 ± 14.73	26.41 ± 11.35	33.72 ± 16.12	.073
UPDRS‐III (challenged)	19.37 ± 8.94	21.50 ± 10.73	17.91 ± 7.32	.149
Improvement rate	0.33 ± 0.17	0.19 ± 0.09	0.44 ± 0.14	<.001
MMSE	25.15 ± 3.5	25.09 ± 3.21	25.18 ± 3.74	.922
H&Y	2 (1.5, 2.5)	2 (1.75, 2.5)	2.0 (1.5, 2.5)	.45
ROTAP	0.31(0.10,0.63)	(0.31 (0.10, 0.63)	(0.31 (0.10, 0.63)	.911

Abbreviations: H&Y, Hoehn and Yahr staging; MMSE, mini‐mental state examination; NA, not applicable; PD, Parkinson's disease; UPDRS, Unified Parkinson's Disease Rating Scale; ROTAP, ratio of mean tremor score and mean PIGD score.

### 3.2 TBSS identified white matter damage in corpus callosum, corona radiata, and internal capsule

**FIGURE 2:**
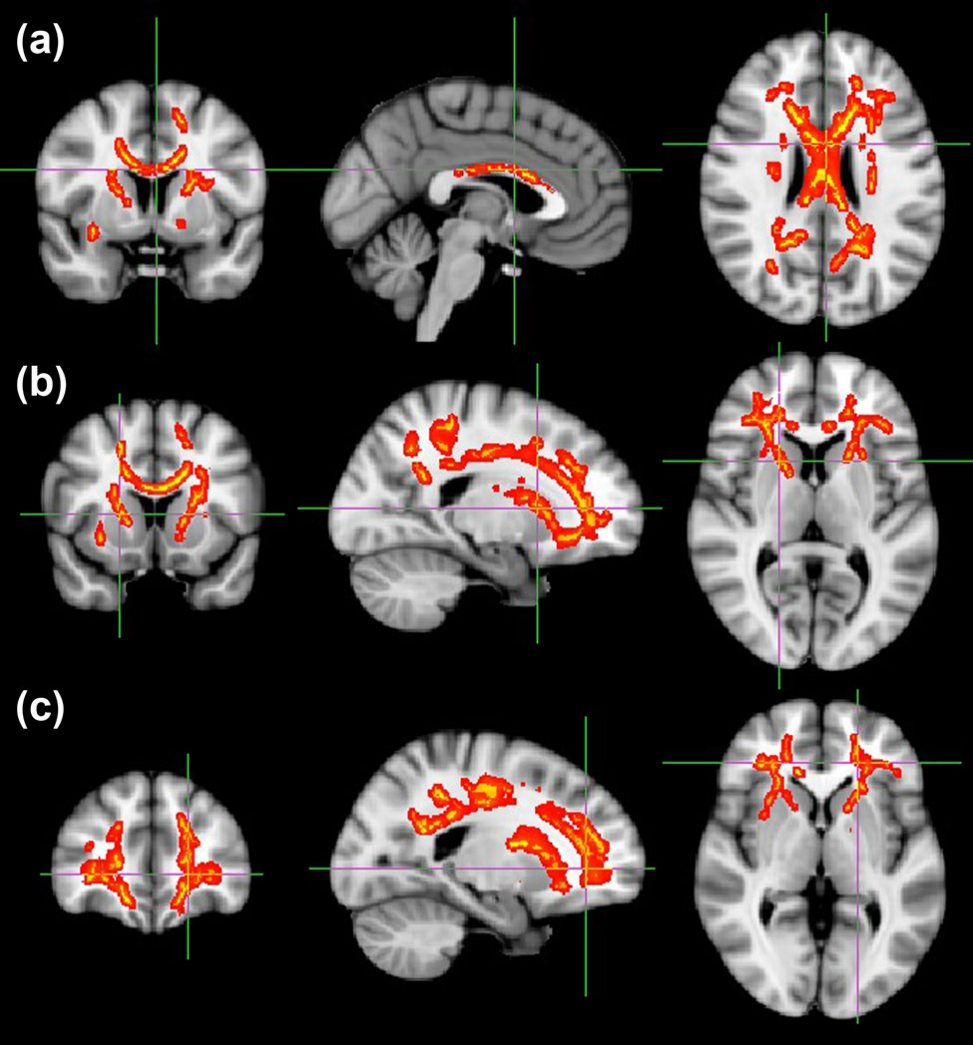
Significant decrease of fractional anisotropy (FA) was found in brain areas of patients in the levodopa irresponsive group compared to the responsive group with age, sex, baseline UPDRS‐III score, challenged UPDRS‐III score, MMSE score, and H&Y stage adjusted as covariates. (A) splenium/body/genu of the corpus callosum;(B) bilateral limb of internal capsule;(C) bilateral anterior /superior corona radiata

**FIGURE 3:**
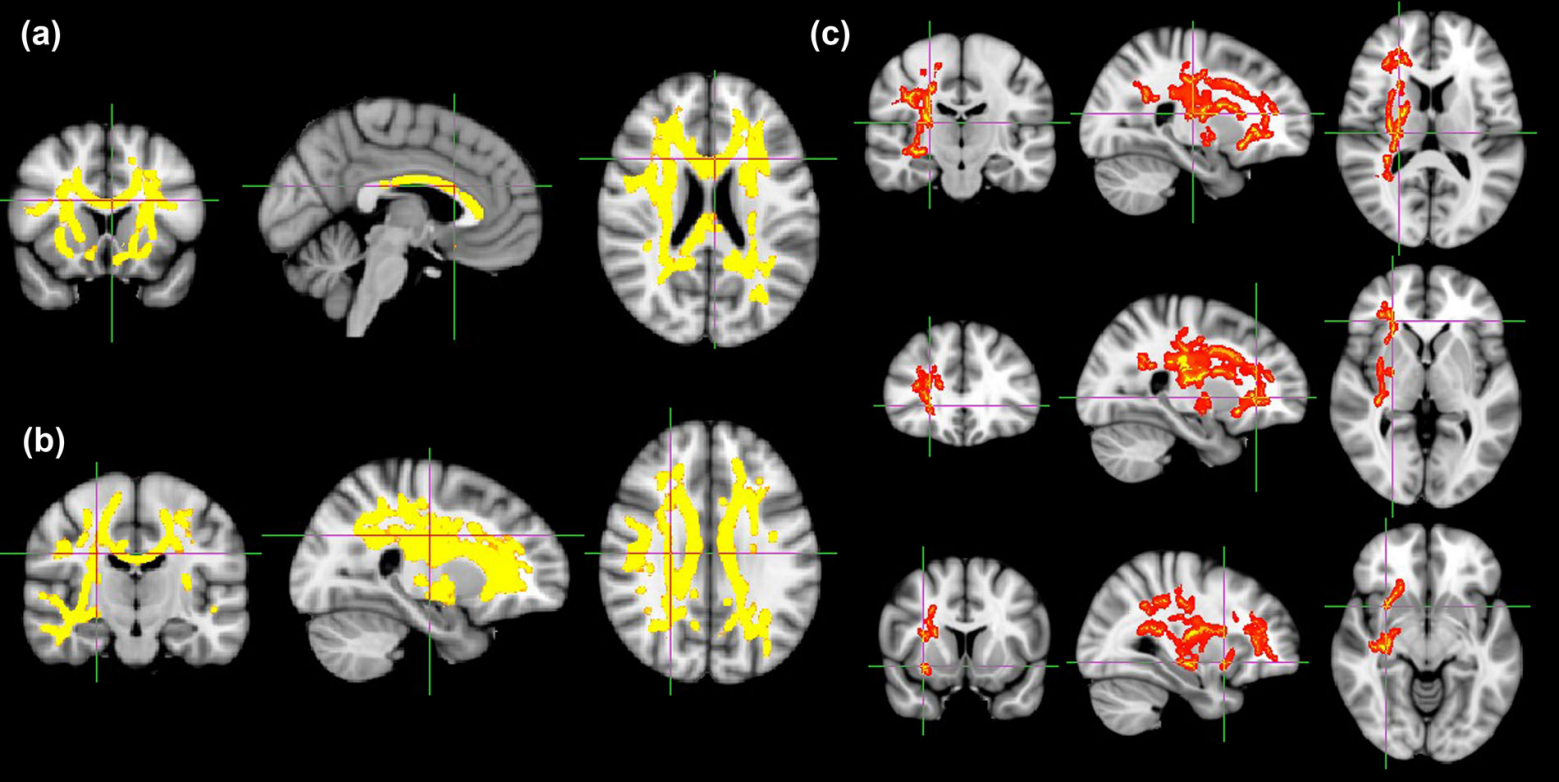
Significant increase in mean diffusivity (MD), axial diffusivity (AD), and radial diffusivity (RD) was found in the brain areas of patients in levodopa irresponsive group compared to the responsive group. (A) wide‐ranging brain WM areas of MD.(B) wide‐ranging brain WM areas of RD. (C)main brain region of AD. From the top to bottom, they are the right posterior limb of internal capsule, anterior/ superior/ posterior corona radiata, external capsule

Compared with the responsive group, patients in the irresponsive group exhibited significantly reduced FA in the splenium/body/genu of the corpus callosum, bilateral limb of the internal capsule, bilateral anterior/superior corona radiata (TFCE‐corrected *p* < .05) (Figure 2). The irresponsive group had a higher MD (TFCE‐corrected *p* < .05) and RD (TFCE‐corrected *p* < .05) (Figure 3) in wide‐ranging brain WM areas than the responsive group; The irresponsive group had a higher AD in the right posterior limb of internal capsule, anterior/superior/posterior corona radiata and external capsule (TFCE‐corrected *p* < .05) (Figure 3). However, we did not identify any region in which the metrics are positively or negatively related to the improvement rate. Among the four metrics, only FA presented nonsignificant negative relation with an improvement rate in a small region covering parts of the genu of the corpus callosum, body of corpus callosum, left anterior corona radiata, and cingulum (TFCE‐corrected *p* < 0.25) (Figure S2).

### 3.3 DT and ROI analysis identified FA decrease in the corpus callosum

Comparing the mean FA, MD, AD, and RD of the corpus callosum fiber we had tracked, the DT showed the irresponsive group had reduced FA compared with the responsive groups (0.56 ± 0.02 vs. 0.57 ± 0.02, *p* = .032). No statistically significant differences were observed in mean MD (0.0009 ± 0.00005 vs. 0.0008 ± 0.00004, *p* = .12, AD (0.0016 ± 0.00007 vs. 0.0015 ± 0.00007, *p* = .397), RD (0.00059 ± 0.00005 vs. 0.00058 ± 0.00007, *p* = .536) between the irresponsive group and responsive group (Figure 1). The ROI analysis showed the irresponsive group had reduced FA (0.58 ± 0.02 vs. 0.60 ± 0.02, *p* = .003) and decreased RD (0.00039 ± 0.00015 vs. 0.00038 ± 0.00017, *p* = .017) compared with the responsive groups. No statistically significant differences were observed in mean MD (0.0003 ± 0.00015 vs. 0.0003 ± 0.00016, *p* = .053, AD (0.0014 ± 0.00016 vs. 0.0014 ± 0.00017, *p* = .413), between the irresponsive group and responsive group (Figure 1). Notably, the results of DT and ROI analyses on corpus callosum may only reflect an averaged difference along the whole tract, and potentially more significant differences may be glossed over.

### 3.4 AFQ found no difference in fiber tracts between the two groups

Before FDR correction, the irresponsive group showed FA reduction relative to the responsive group in the anterior portion of the bilateral thalamic radiation, CST, left arcuate, the posterior portion of bilateral cingulum cingulate, right IFOF, right uncinate, callosum forceps major, the scattered portion of callosum forceps minor and bilateral superior longitudinal fasciculus. The irresponsive group showed MD, AD, and RD increased relative to the responsive group in many regions of the 20 fiber tracts (flowchart and complete results are shown in Figure S3). However, after FDR correction, no statistically significant FA/MD/AD/RD differences between the irresponsive and responsive groups were noticed (Figures S4–S7).

### 3.5 Brain network analysis

#### 3.5.1 Global network metrics indicate impaired integration function

**FIGURE 4:**
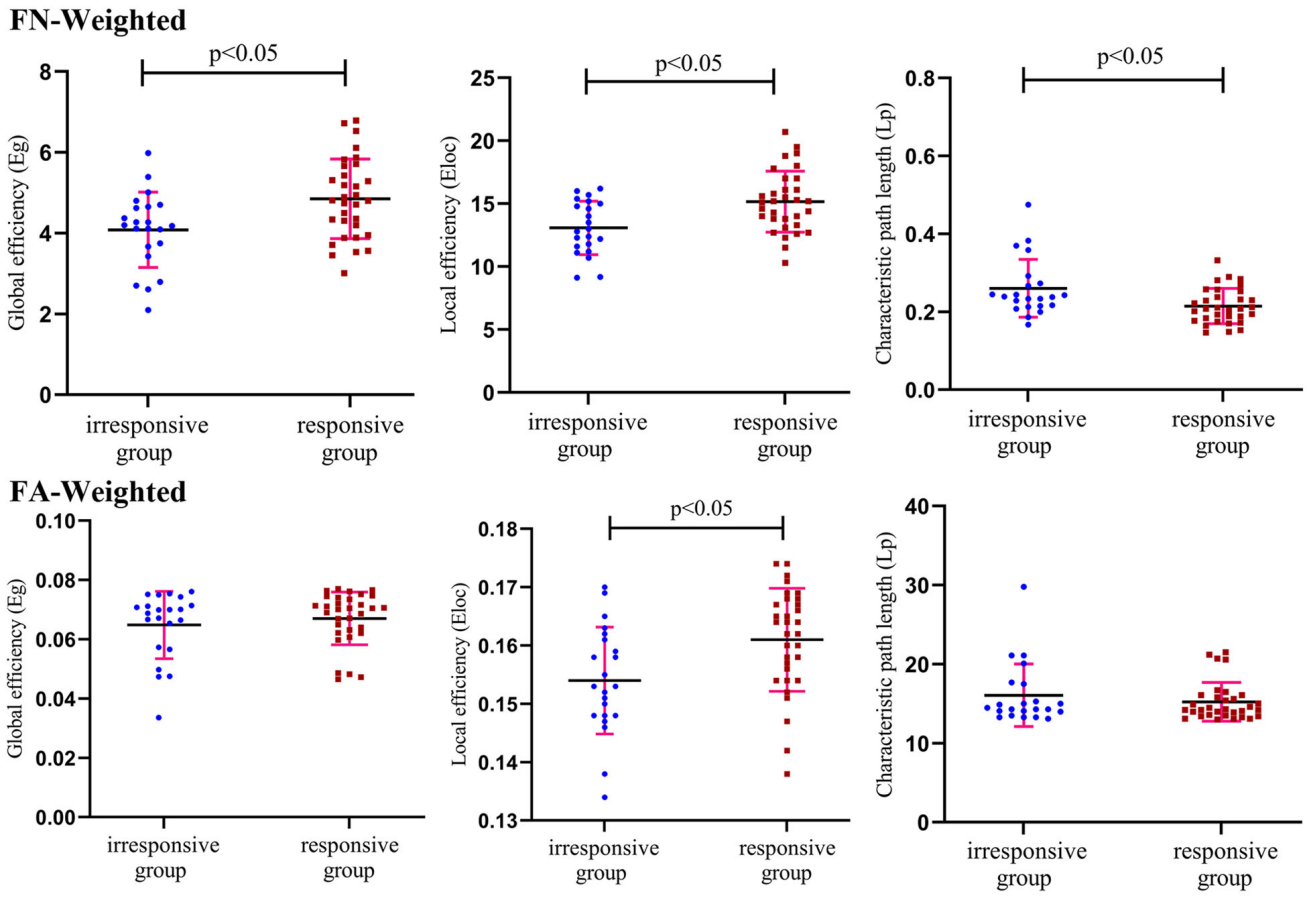
Results of global topological properties. The blue dot indicates the topological index value of the brain network corresponding to each subject in the irresponsive group, and the red dot indicates the responsive group. The middle line segment is the mean value, and the upper and lower line segments are standard deviations

The global network topological metrics of the two groups are shown in Table 2. For the FN‐weighted network, statistical differences were found in the *E*_g_, *E*_loc_, and *L*_p_ between the irresponsive group and responsive group, respectively. There was no significant difference in the clustering coefficient (*C*_p_) between the two groups. For the FA‐weighted network, statistical differences were found in the *E*_loc_ between the irresponsive group and responsive group, respectively. There was no significant difference in *E*_g_, *C*_p_, and *L*_p_ between the two groups (Figure 4).

**TABLE 2:** Comparison of global network topology difference among two groups

Network topological metrics	FN‐Weighted			FA‐Weighted		
	Irresponsive group	Responsive group	*p*‐values	Irresponsive group	Responsive group	*p*‐values
*E*_g_	4.08 ± 0.91	4.85 ± 0.95	*.005	0.07 ± 0.01	0.07 ± 0.01	.40
*E*_loc_	13.07 ± 2.07	15.16 ± 2.4	*.002	0.15 ± 0.01	0.16 ± 0.009	*.007
*L*_p_	0.26 ± 0.07	0.21 ± 0.04	*.007	16.06 ± 3.84	15.23 ± 2.41	.30
*C*_p_	0.05 ± 0.01	0.06 ± 0.01	.17	0.21 ± 0.03	0.16 ± 0.03	.63
Σ	9.12 ± 1.15	9.31 ± 1.33	.60	8.64 ± 1.20	11.85 ± 2.25	.83
Λ	1.59 ± 0.25	1.54 ± 0.23	.44	1.47 ± 0.28	0.21 ± 0.04	.81
Γ	14.27 ± 1.27	14.07 ± 1.35	.60	12.43 ± 0.74	14.80 ± 3.30	.50

Abbreviations: Eg, global efficiency; Eloc, local efficiency; Lp, characteristic path length; Cp, clustering coefficient; σ, small‐worldness; λ, normalized characteristic path length;γ, normalized clustering coefficient.

#### 3.5.2 Both groups showed small‐worldness

Patients in both irresponsive and responsive groups showed small‐worldness in FN‐weighted network and FA‐weighted network attributes with a larger clustering coefficient (γ > > 1) and similar characteristic path length (λ≈1). There was no significant difference in small‐worldness (σ), normalized clustering coefficient (γ), and normalized characteristic path length (λ) between the two groups, respectively (Table 2).

#### 3.5.3 No difference between nodes and edges analysis

There was no significant difference in nodal network topological metrics, including betweenness centrality, degree centrality, nodal clustering coefficient, nodal efficiency, and nodal local efficiency (Table S1). There was no difference in edge connective strength between the two groups either in the FN‐weighted network or the FN‐weighted network using a two‐sample *t*‐test after FDR correction. After NBS correction, there was no significant difference between the two groups.

## 4 DISCUSSION

In this study on PIGD subtype PD patients, we identified WM alterations characterized by decreased FA in the splenium/body/genu of the corpus callosum, bilateral limb of internal capsule, bilateral anterior/superior corona radiata, left posterior thalamic radiation, increased AD in the right posterior limb of internal capsule, anterior/superior/posterior corona radiata and external capsule, accompanied by a wide‐spread increase of MD and RD surrounding corpus callosum, in the irresponsive group compared with the responsive group. More specifically, we further analyzed the alterations in corpus callosum using DT and ROI‐based analyses and found decreased FA in patients of the irresponsive group compared with the responsive group, while no difference in MD, AD, and RD was identified between these two groups. We also used AFQ method to analyze the WM changes in 20 WM tracts between the two groups but failed to identify any statistically significant difference. In TBSS analysis, a wide‐spread increase of MD and RD surrounding corpus callosum may suggest possible damage in myelin and broad cellular damages (Zhan et al., 2012), while decreased FA suggests possible damage in axonal integrity, and the degree of axonal myelination of WM (Zhang & Burock, 2020) in relatively more limited and specific regions compared with MD and RD. Regions with increased AD partially overlapped the regions with decreased FA, which may also indicate damage in myelin (Alexander et al., 2007; Zhan et al., 2012). Therefore, our findings in TBSS analysis may suggest that (1) PIGD type PD patients with poor responsiveness to levodopa present possible wide‐spread myelin damage; (2) damage of microstructure of WM exists in the corpus callosum, limb of internal capsule, corona radiata, and left posterior thalamic radiation; (3) increase of AD in the limb of internal capsule, corona radiata, and external capsule suggest severer myelin damage in these areas. Interestingly, regions with decreased FA partially overlapped regions with increased AD, and these overlapped regions were also covered by regions with increased MD and RD, suggesting that WM damage involving myelin and microstructure was more prominent in limb of internal capsule and corona radiata. Previous pathological findings suggested that the pathogenic forms of α‐synuclein can be transported along axons (Peng et al., 2020); WM alterations might be a consequence of GM damage or representation of α‐synuclein pathological spreading between anatomically connected brain areas (Peng et, al., 2020, Sarasso et al., 2021, Van Den Berge et al., 2021; Zhang & Burock, 2020). Notably, the different areas in this study, internal capsule and corona radiata are both known as regions with high density of axons. We speculate that the WM damage in internal capsule and corona radiata in PD patients with poor responsiveness to levodopa may result from damage in grey matter due to α‐synucleinopathy. Therefore, it is reasonable to assume that PD patients with severer damage in grey matters, especially striatum, substantia nigra, and thalamus, may have poorer responses to levodopa (Delva et al., 2020), which may be reflected by WM damage in internal capsule and corona radiata. However, more direct evidences are needed to support this speculation. Moreover, dopamine synthesized in substantia nigra is transported in four major dopaminergic pathways, including the mesocortical pathway, mesolimbic pathway, nigrostriatal pathway, and tuberoinfundibular pathway (Björklund & Dunnett, 2007; Klein et al., 2019). WM damage in the internal capsule and corona radiata may disrupt three of these four pathways (mesocortical pathway, mesolimbic pathway, and nigrostriatal pathway), and thus lead to poor responsiveness to levodopa. Previous neuroimaging studies have shown that dopaminergic depletion in PD is accompanied by dysfunction of the basal ganglia motor circuit (Haslinger et al., 2001), resulting in hypoactivation of the supplementary motor area and striatum as well as decreased connectivity of the striato‐thalamo‐cortical motor pathways. Levodopa treatment has been reported to normalize the function of the basal ganglia motor circuit and restore striato‐cortical motor pathway connectivity, striato‐thalamo‐cortical and subthalamic nucleus‐cortical pathways in a manner associated with improvements in motor function (Agosta et al., 2014; Gao et al., 2017; Kwak et al., 2010). Therefore, damage in internal capsule and corona radiata may disrupt the striato‐thalamo‐cortical and subthalamic nucleus‐cortical pathways, resulting in poor levodopa responsiveness.

Additionally, decreased FA was identified in splenium/body/genu of the corpus callosum in patients in the irresponsive group compared with the responsive group in TBSS analysis, DT, and ROI analysis. WM damage in the corpus callosum has been identified in many studies on PD (Zhou et al., 2021), it could occur in newly‐onset PD patients, and get severer as PD progresses (Wen et al., 2018). Damage in the corpus callosum was also known to be more obvious in PIGD‐type PD patients with lower FA and higher RD, compared with tremor‐dominant type PD patients (Wen et al., 2018). However, no previous study investigated the relationship between corpus callosum damage and levodopa responsiveness in PD patients. In our study, there is decreased FA without significant difference in AD, MD, and RD of the corpus callosum in the irresponsive group compared with the responsive group. No significant difference in MD, AD, and RD may be explained because high MD is thought to indicate broad cellular damages including edema and necrosis, while levodopa responsiveness is related to neuron degeneration. RD appears to describe myelin pathology‐induced myelin thinning, and changed AD indicates acute axonal injury, while PD is a chronic progressive neurodegenerative disease. Therefore, we speculate that the damage to the corpus callosum is mainly due to axonal degeneration and demyelination. The WM fibers lesion burden was found to be possibly associated with the impairments of fiber‐connected gray matter regions; furthermore, WM damage may also represent the spread of α‐synucleinopathy along axons in PD patients (Sarasso et al., 2020; Van Den Berge et al., 2021). Corpus callosum contains more than 200 million commissural fibers connecting the two hemispheres and is by far the largest WM fiber bundle in the human brain (Wang et al., 2021). We speculate that corpus callosum damage may suggest more prominent gray matter damage in both cerebral hemispheres and a wider spread of α‐synuclein in PD patients with poor responsiveness to levodopa. Moreover, corpus callosum serves as a crucial organization for information integration between the two hemispheres (Wang et al., 2021), and plays an integral role in the interhemispheric transfer of sensory, motor, and higher order cognitive functions (Gazzaniga, 2000), Damage in corpus callosum may disrupt information integration of the whole brain network, which may affect the programming and regulation of movement, and also may affect the cortex‐regulated phasic dopamine release in substantia nigra pars compacta, resulting in poor levodopa responsiveness.

Furthermore, we also explored the potential relationship between levodopa responsiveness and damage of other WM tracts using AFQ methods, and we found that no significant damage of these 20 WM tracts was related to levodopa responsiveness after FDR correction. It is possible that AFQ may divide the fibers into excessively small segments, resulting in less possibility of difference. Moreover, the limitation of AFQ is that fiber tracts may not follow the true brain information transfer pathways because of limitations of tractography parameters (Yeatman et al., 2012). However, we found that before FDR correction, bilateral cingulate, left arcuate fasciculus, right IFOF, right uncinate, and bilateral superior longitudinal fasciculus trend to present lower FA in the irresponsive group compared with the responsive group. These findings in AFQ suggest that levodopa responsiveness‐related damages potentially exist in the main WM tract in PD patients. Although these damages are not statistically significant, they need a detailed investigation.

Interestingly, we did not find any region presenting a positive or negative relationship between the four metrics and the improvement rate using linear regression. We speculate that this lack of relationship may be explained by the threshold theory of PD (Engelender & Isacson, 2017). According to the threshold theory, symptoms of PD occur when its pathology spreads and neuronal damage accumulates to a threshold level. Similarly, it is likely that a threshold of levodopa responsiveness may also exist. That is, levodopa treatment provides material for dopamine synthesis for dopaminergic neurons (Lewitt, 2008), when the damage in neurons and brain network reaches the threshold by which dopaminergic neurons could not synthesize sufficient dopamine for sustaining the function of the whole brain network, the levodopa responsiveness may decrease precipitously, leading to more convincing findings. However, direct evidence is needed to support our speculation.

The modality of whole‐brain information flow can be conceptualized as the structural brain network. In the FN‐weighted network, decreased *E*_g_ and increased *L*_p_ were observed in the irresponsive patients in our study compared with responsive patients, reflecting decreased efficiency of the whole brain network and disruption of network information integration. Meanwhile, in both FN‐ and FA‐weighted networks, decreased *E*_loc_ in irresponsive patients indicates damage in segregation of the structural brain network.

Integration of the structural brain network refers to specific information transmission and exchange between distributed areas (Rubinov & Sporns, 2010). Impairments of integration suggest the presence of reduced efficiency and extra costs of information transmission between functional areas in the irresponsive group (Li et al., 2017). Altered information exchange among the whole brain may account for poor responsiveness to levodopa. Interestingly, the FN‐weighted network showed decreased *E*_g_ and increased *L*_p_ in irresponsive group compared with responsive group, while FA‐weighted network did not show such alteration, which may suggest that the damage of the network in irresponsive PD patients may more likely be attributed to the decreased number of fibers rather than microstructure of WM damage. It is possible that severer WM damage which causes a decrease in fiber number, rather than relatively mild damage of WM such as FA decrease‐reflected microstructural damage, accounts for the disruption of network integration in irresponsive PD patients, and the accumulation of microstructure damage may lead to the decreased number of fibers, Therefore, PD patients may present better responsiveness to levodopa at an early stage, and this responsiveness worsens as PD progresses and WM damage worsens (Wen et al., 2018).

In the present study, both the irresponsive and responsive groups presented favorable small‐worldness. No difference in nodal network topological metrics or edge connective strength was found. This finding indicates that the network damage in irresponsive PD patients is more likely to be a global damage of the WM network, rather than damage in some specific brain regions or damage of connection between specific brain regions. Therefore, when investigating levodopa responsiveness in PD patients, it may be reasonable to consider the damage to the whole brain. However, damage in some specific brain regions is well‐known in PD (e.g., substantia nigra), and analysis of these regions may also be helpful.

Notably, the analytical methods used in this study, namely TBSS, DT, ROI analysis, and AFQ, have different features, and may present different results on the same item. For instance, TBSS and AFQ need multiple comparison corrections, and thus have the risk of overcorrection. TBSS is a WM skeleton‐based voxelwise analytical method, DT and AFQ are fiber tract‐based analyses, while ROI analysis is region‐based analysis, which may bring different biases. DT and ROI analysis average the parameters along the tract or inside the region, AFQ segments each tract into multiple parts, while TBSS compares parameters in each voxel, which may also bring different biases.

More importantly, due to the limit of scanning equipment, an acquisition protocol of 15 noncollinear directions with *b* = 800 s/mm^2^ was chosen in this study. It has been reported that acquisition protocol of < 30 noncollinear directions with *b* < 1000 s/mm^2^ may lead to a greater spread of the fiber‐tracking results, lower power to resolve crossing fibers, and noisier streamlines when using a probabilistic fiber‐tracking approach (Calamante, 2019). However, for DT, the number of noncollinear directions of 11, 15, 21, and 31 orientations present similar tracking results (Yao et al., 2015), while *b*‐value ≤ 800 s/mm^2^ is also used in many studies and has been proven to present favorable results of tractography (Hana et al., 2014). Moreover, it has been reported that fewer directions have a greater impact on probabilistic tracking, rather than DT (Soares et al., 2013), while all methods we used, including DT, ROI analysis, AFQ, and brain network analysis, are based on DT. Therefore, we chose a relatively suitable, although not ideal, acquisition protocol based on the limit of the equipment. Anyway, the accuracy of tractography in our study should be considered judiciously.

Several limitations exist in this study. First, the number of included PD patients was limited, so these findings need to be validated in a larger population. Second, only 15 noncollinear directions were applied to DTI scans in this study, which may limit the accuracy of reconstruction. Third, we analyzed the structural brain network based on DTI. It is generally known that the ability to resolve crossing fibers is limited in present DTI processing. Finally, as a cross‐sectional study, these results could not reflect the relationship between the exacerbation of levodopa responsiveness and changes in WM impairment and brain network.

## 5 CONCLUSIONS

This study indicates that PD patients with poor responsiveness to levodopa had WM damage in multiple brain areas, which may cause disruption of integration of the structural brain network. More specifically, damage in the corpus callosum was obvious in PD patients with poor responsiveness to levodopa. This impairment of WM and brain network may serve as a potential neuroimaging marker for the evaluation of dopamine replacement therapy for PD and may provide insights into the mechanism of levodopa resistance in some PD patients.

## AUTHOR CONTRIBUTIONS

Conception and design: JD, XL, and LC; acquisition of data: JD, XZ, CD, HL, and LZ; analysis and interpretation of data: JD, XL, and YZ: drafting the article: JD, LM, and XL; critically revising the article: XL, CT, and LC; revision of submitted version of manuscript: JD, XZ, CD, LZ, YL, YZ, HL, GL, LM, CT, XL, LC; approved the final version of the manuscript on behalf of all authors: LC; statistical analysis: JD, YL, and GL; administrative/technical/material support: XL and LC; supervision: LC. All authors contributed to the article and approved the submitted version.

## CONFLICT OF INTEREST

The authors declare that they have no conflicts of interest.

### PEER REVIEW

The peer review history for this article is available at https://publons.com/publon/10.1002/brb3.2825.

## Supporting information

Table S1

Figure S1–S7

## Data Availability

All data used and generated in this work is available under reasonable request to the corresponding authors.
