# Supplementary material for: Levodopa responsiveness and white matter alterations in Parkinson's disease: A DTI‐based study and brain network analysis: A cross‐sectional study
Source: Brain Behav. 2022 Nov 24;12(12):e2825. doi: 10.1002/brb3.2825 (PMC9759147; doi:10.1002/brb3.2825)
Supplement: Supplementary file 2 — Figure S1–S7 [file BRB3-12-e2825-s001.pdf]

Levodopa Responsiveness and White Matter Alterations in Parkinson's Disease: A DTI-Based Study and Brain Network Analysis: A Cross-Sectional Study

Juncong Du[1], M.D., Xuan Zhou[1], M.D, Yi Liang[1], M.D., Lili Zhao[1], M.D., Chengcheng Dai[1], M.D., Yuke Zhong[1], M.D., Hang Liu[1], M.D., Guohui Liu[1], M.D. Lijuan Mo[1], M.D., Changhong Tan[1], Ph.D, M.D., Xi Liu[1, *], PhD, M.D., Lifen Chen[1, *], Ph.D, M.D.

1 Department of Neurology, The Second Affiliated Hospital of Chongqing Medical University

Supplementary

eFigure 1. Mean matrixes of different type and group.
eFigure 2. TBSS result of relation between FA and levodopa responsiveness.
eFigure 3. Flowchart of AFQ for Pointwise Comparison.
eFigure 4. Representative tract of AFQ, and AFQ results of FA
eFigure 5. AFQ results of MD
eFigure 6. AFQ results of RD
eFigure 7. AFQ results of AD

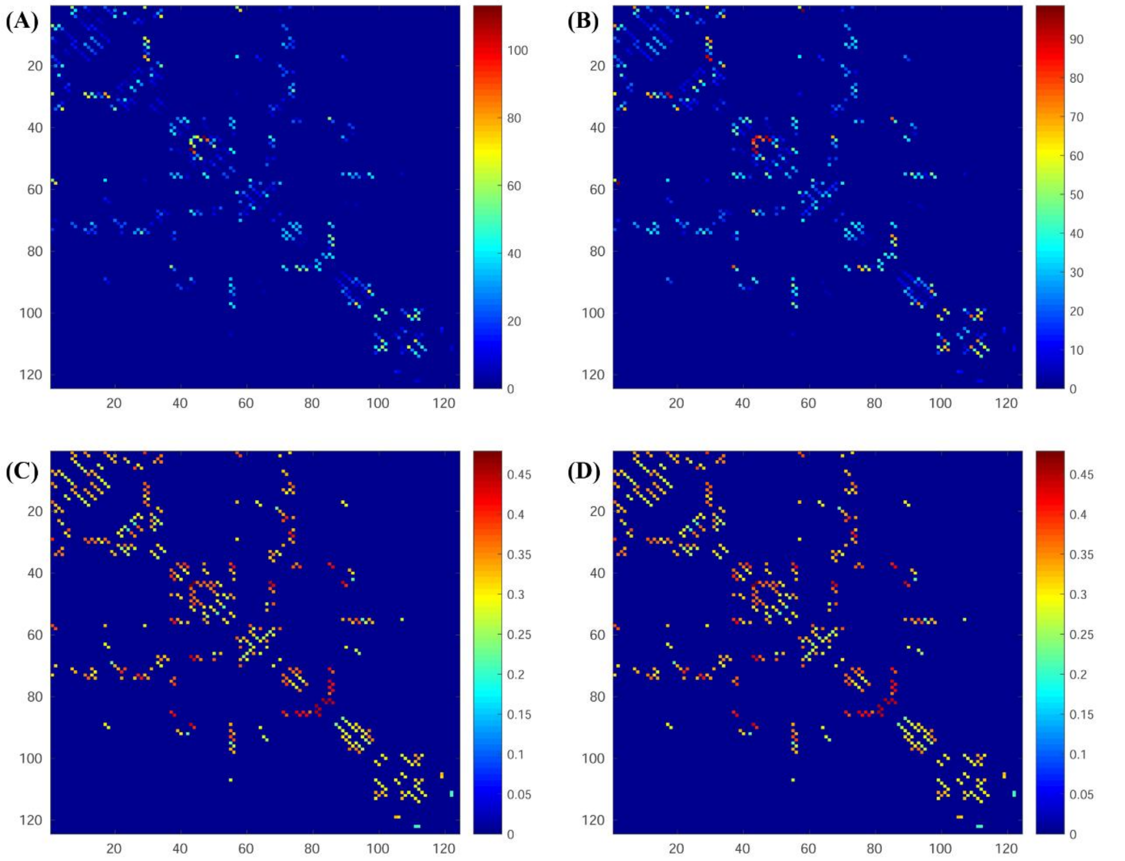

eFigure.1 Mean matrixes. (A) the mean FN-weighted network matrixes of irresponsive group; (B) the mean FN-weighted network matrixes of responsive group. (C) the mean FA-weighted network matrixes of irresponsive group; (D) the mean FA-weighted network matrixes of responsive group.

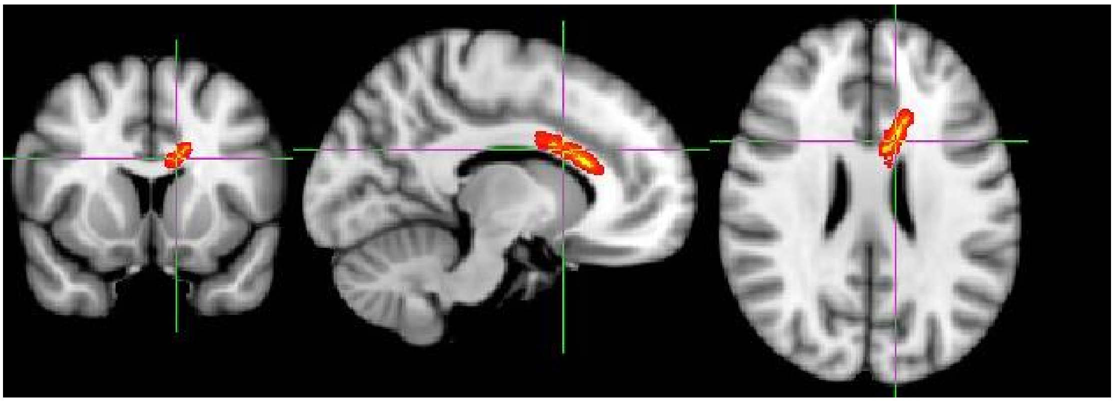

eFigure.2 TBSS result of relation between FA and levodopa responsiveness. The FA of regions labeled with red-yellow color is negatively related with improvement rate in acute levodopa challenge test but without statistical significance (TFCE-corrected p<0.25), which covers parts of genu of corpus callosum, body of corpus callosum, left anterior corona radiata, and cingulum.

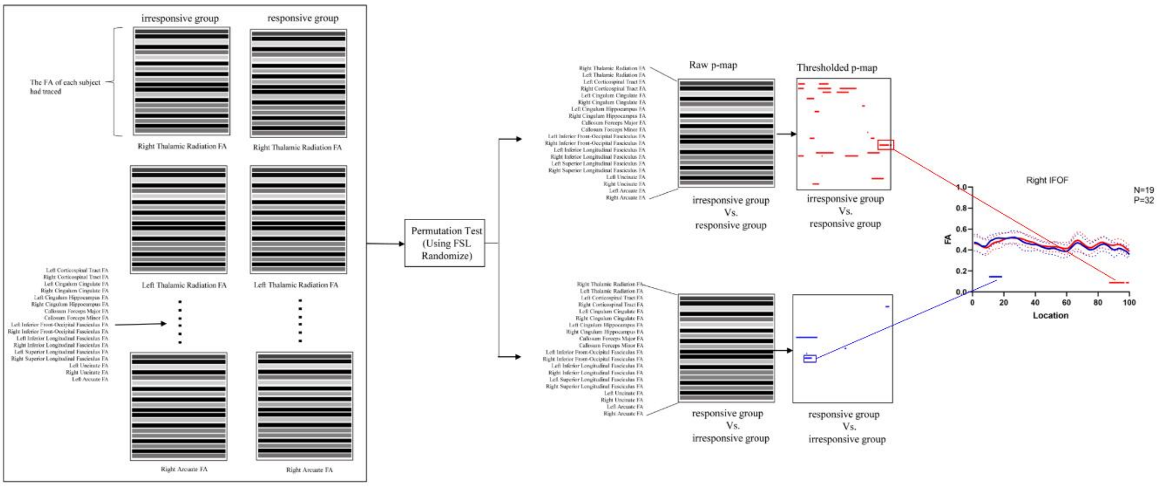

eFigure.3 Flowchart of AFQ for Pointwise Comparison. Each tract profile from all subjects were arranged in tract-based matrices. All these matrixes were fed into permutation-based statistical analysis with 5000 permutations using the FSL Randomize program. After False discovery rate (FDR) correction, no significant difference was identified. The statistical results thresholded by P<0.05 and finally displayed as bars under each tract profile plot.

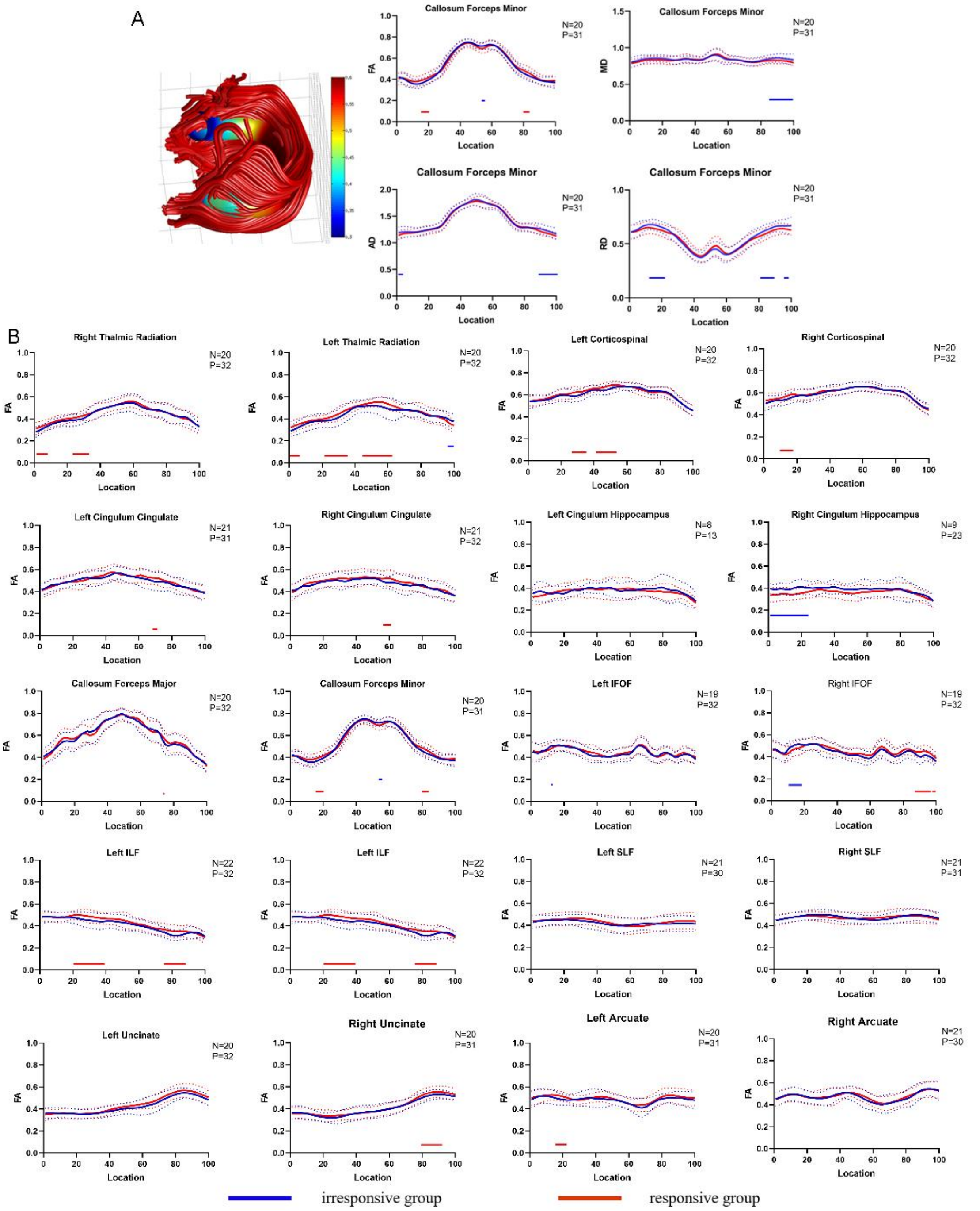

eFigure.4 (A) Callosum forceps minor as the representative tract of AFQ, and the plots of its FA, MD, AD, RD profiles. (B) The plots of FA profiles of 20 identified fiber tracts from irresponsive group and responsive group. (Blue for irresponsive group, red for responsive) in mean (SD) (solid lines for means and dotted line for SDs). The red bars under the FA profile indicate the regions of significant difference of decreased FA compared irresponsive group to responsive group; the blue bars under the FA profile indicate the regions of decreased FA compared responsive group to irresponsive group. The x-axis represents the location between the beginning and termination waypoint regions of interest.

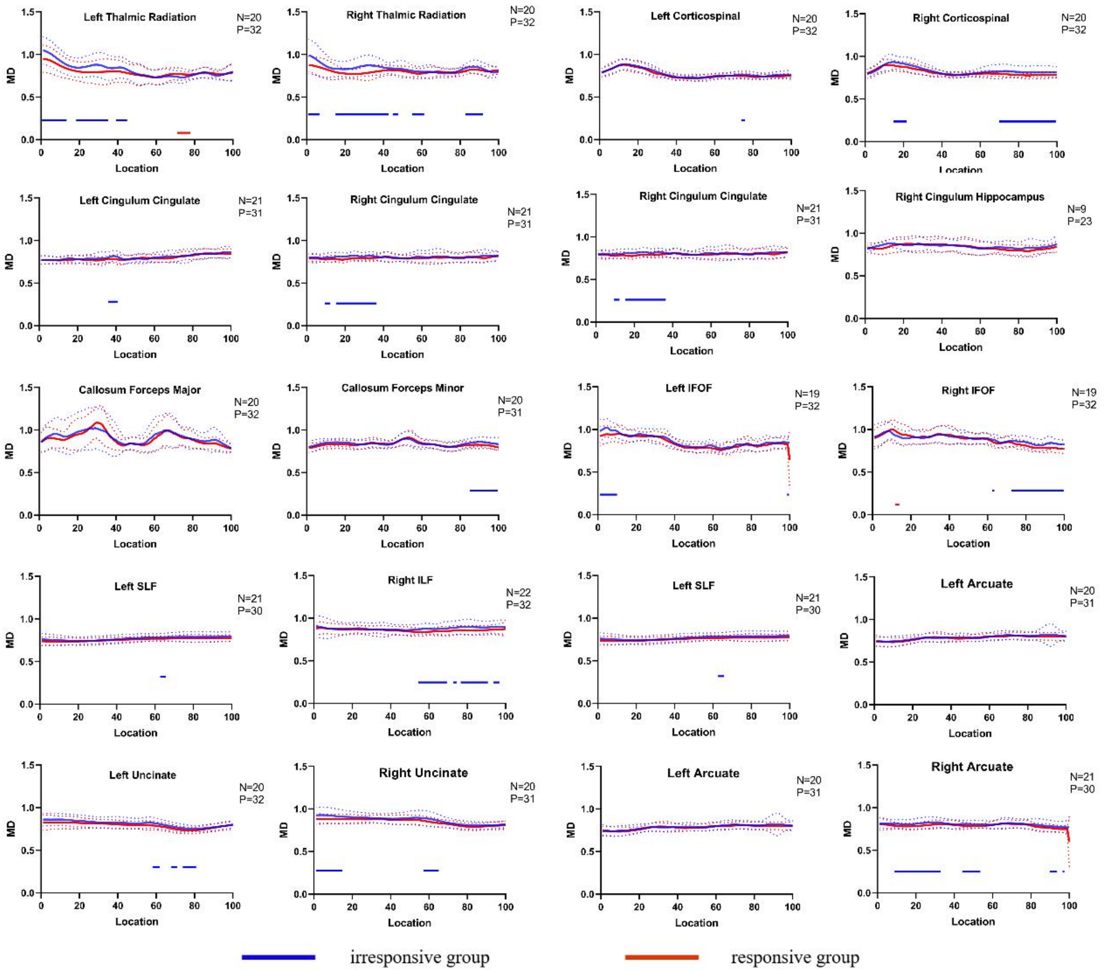

eFigure.5 The plots of MD profiles of 20 identified fiber tracts from irresponsive group and responsive group (Blue for irresponsive group, red for responsive) in mean (SD) (solid lines for means and dotted line for SDs). The red bars under the MD profile indicate the regions of significant difference of decreased MD compared irresponsive group to responsive group; the blue bars under the MD profile indicate the regions of decreased MD compared responsive group to irresponsive group. The x-axis represents the location between the beginning and termination waypoint regions of interest.

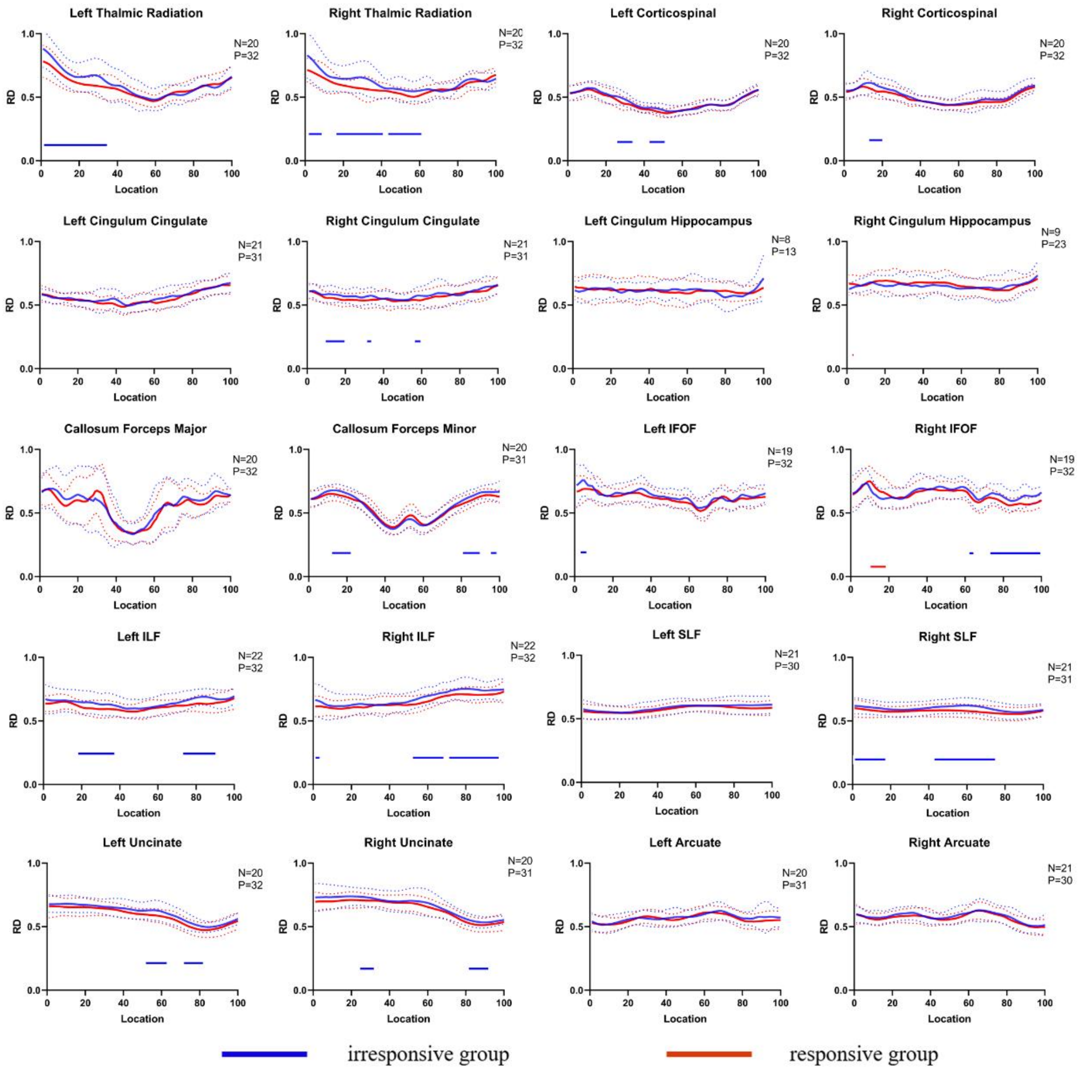

eFigure.6 The plots of RD profiles of 20 identified fiber tracts from irresponsive group and responsive group (Blue for irresponsive group, red for responsive) in mean (SD) (solid lines for means and dotted line for SDs). The red bars under the RD profile indicate the regions of significant difference of decreased RD compared irresponsive group to responsive group; the blue bars under the RD profile indicate the regions of decreased RD compared responsive group to irresponsive group. The x-axis represents the location between the beginning and termination waypoint regions of interest.

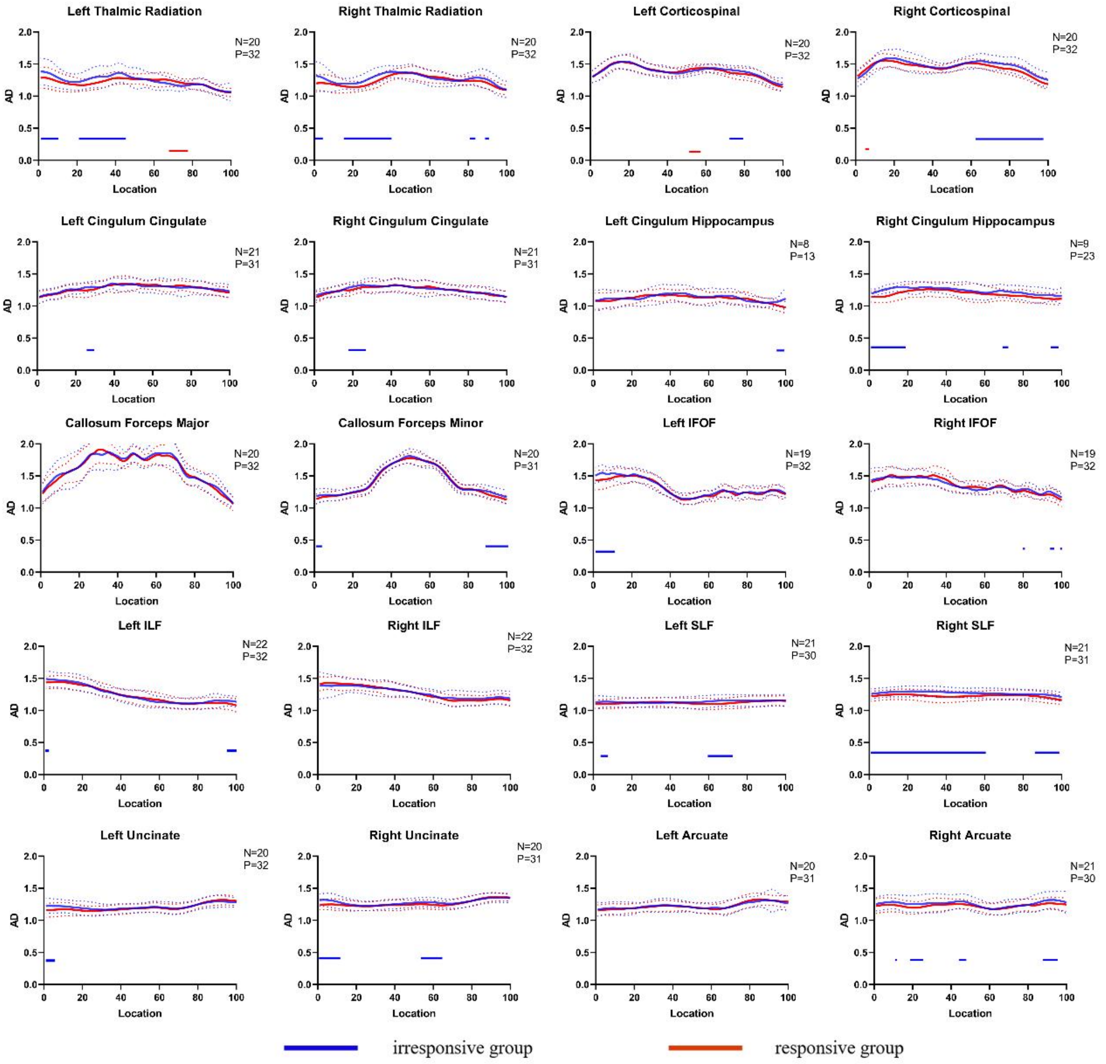

eFigure.7 The plots of AD profiles of 20 identified fiber tracts from irresponsive group and responsive group (Blue for irresponsive group, red for responsive) in mean (SD) (solid lines for means and dotted line for SDs). The red bars under the AD profile indicate the regions of significant difference of decreased FA compared irresponsive group to responsive group; the blue bars under the AD profile indicate the regions of decreased AD compared responsive group to irresponsive group. The x-axis represents the location between the beginning and termination waypoint regions of interest.
